# A Comprehensive Characterization of Biodegradable Edible Films Based on Potato Peel Starch Plasticized with Glycerol

**DOI:** 10.3390/polym14173462

**Published:** 2022-08-25

**Authors:** Albert Linton Charles, Nomalungelo Motsa, Annur Ahadi Abdillah

**Affiliations:** 1 Department of Tropical Agriculture and International Cooperation, National Pingtung University of Science and Technology, 1 Shuefu Road, Neipu, Pingtung 91201, Taiwan; 2 International Master Degree Program in Food Science, National Pingtung University of Science and Technology, 1 Shuefu Road, Neipu, Pingtung 91201, Taiwan; 3 Department of Marine, Faculty of Fisheries and Marine, Universitas Airlangga, Campus C UNAIR, Mulyorejo, Surabaya 60115, Indonesia

**Keywords:** biodegradable edible film, food waste, glass transition, green product, plastic debris, thermoplastic starch

## Abstract

Potatoes are a source of starch, which is an eco-friendly alternative to petrochemicals in plastic production. Increasing potato production also creates agricultural waste that could be converted to potato peel starch (PPS) and developed as films. A response surface method approach was employed to optimize the bioconversion of PPS (2, 4, and 6% *w*/*v*) and compared with carboxymethyl cellulose (CMC)-based films. The microstructure analysis of PPSF showed increased thickness, decreased swelling power, water solubility, and vapor permeability, which were linked to increased molecular interactions as a function of PPS increments. However, low-starch PPSF exhibited high transparency, good mechanical properties, and thermal stability (high melting temperature), pliability, and accelerated seawater and soil biodegradation (~90%: 20 and 50 days, respectively). All films exhibited thermal stability at >100 °C and retained similar amorphous characteristics, evidenced by their flexibility, which confirmed the potential use for PPS in packaging perishable and cooled foods.

## 1. Introduction

Food security is a global issue, particularly in developing countries; nonetheless, and according to Ref [1], approximately 1.3 billion of the 6 billion metric tons of food produced annually is wasted mainly between agricultural production and consumption, which highlights the inefficiencies of the entire food chain system [2]. Hence, reducing food loss and waste has gained precedence in the efforts to mitigate global hunger and resource waste, which has been incorporated in the United Nations sustainable development goals (SDG) under SDG 12, with the call to “reduce per capita global food waste at retail and consumer levels and reduce food loss along production and supply chains, including post-harvest loss by 2030” [3].

The widespread use of petroleum-based plastics by the food packaging industry has inevitably led to damages to the niche areas of the environment, such as landfills and oceans, where they concentrate and either fail to degrade, are non-renewable, and possibly insert hazardous chemicals [4]. Consequently, biodegradable (food packaging) films are projected as alternative packaging materials to mitigate the problems related to length of stay and degradation of plastics in land and marine environments [5]. Furthermore, various studies have indicated the usefulness of biodegradable packaging material designed to preserve multiple food products [6], which includes reducing food losses and waste, particularly perishable foods [7].

Natural polymers, such as starch, protein, and lipids, have been investigated in developing biodegradable edible films. However, starch has attracted much research attention and has the potential to develop biodegradable edible films due to its numerous advantages, which include low cost, renewability, and abundant availability, edibility, aesthetic value, good film-forming capability, and degradability [8,9,10,11]. Starch-based edible films have been developed from food materials, such as rice [12], potatoes [13], casava [14], wheat, and corn starches [15]; nevertheless, debate persists over the relative merits of using land to produce crops for non-food use and influencing the sustainable production of plant-based food packaging materials to replace synthetic polymers in the food industry [16]. Among those starch materials, potato is the third most consumed, and around 156 countries are dependent on potato production [17]; however, increasing the production also creates agricultural waste, such as potato peel. This has resulted in the need to investigate the bioconversion of agricultural waste and novel environmentally friendly materials [18] to biodegradable edible films for the packaging industry.

Annual potato production yields approximately 70 to 140 thousand tons of potato peels, with the bulk of the peel waste dumped in landfills, which harms the environment [19]. Traditionally, potato peels have been recycled to produce low-value animal feed, bio-fertilizer, biogas, and a source of antioxidants [19,20,21]. Moreover, numerous investigations have studied the potential use of potato peel in processing valuable products, such as biodegradable food packaging materials [22,23]. However, the physicochemical properties underlying the water barrier properties of potato peel extractable starch to develop an accelerated biodegradable film remain a novelty. Furthermore, the few studies reporting on the potential of potato peel starch (PPS) in producing edible film are limited to biodegradability and water barrier properties, without investigating the molecular and thermal properties of PPS.

Edible films are produced by mixing with a plasticizer that aims to enhance elasticity-like plastic characteristics. The addition of plasticizers is necessary to produce starch-based films, since these films exhibit low mechanical characteristics, which limits the use of the film in conventional package production [24]. Plasticizers such as glycerol hydrogen bond with starch molecules [25], which results in increased thermal stability and mechanical properties of starch-based films [26,27]. 

Cellulose-based polymers possess good potential as bio-based packaging materials, which is linked to their biodegradability and compatibility with nature [28,29]. For example, carboxymethyl cellulose (CMC) has been reported to be used to form excellent biodegradable film, but the application of CMC has been limited due to poor physical properties [6,30,31]. Nevertheless, the scarce reports on the physicochemical, water barrier, and wettability properties of PPS in edible films motivated this study to develop edible films of different PPS concentrations and compare their characteristics against carboxymethyl cellulose (CMC)-based films. Niu et al. [13] reported that the optimized method using the response surface methodology (RSM) was conducted to investigate the correlation effect of the variable on the physical properties of films. Therefore, the drying temperature, PPS concentration, and glycerol concentration were used as combination variables to determine their effects on film thickness; hence, from the preliminary test (RSM), the levels of PPS film formulation were selected to develop PPS films. The effects of starch concentrations were evaluated to hypothetically improve the physicomechanical properties (visual appearance, thickness, moisture content, water solubility, opacity, microstructure of the films, tensile strength, and elongation at break), water barrier properties (swelling power and water vapor permeability), thermal properties using differential scanning calorimetric (DSC) and thermogravimetric analysis (TGA), and crystallinity properties using X-ray diffraction (X-RD), and to exhibit the recommended accelerated biodegradation behavior compared with CMC-based films.

## 2. Materials and Methods

### 2.1. Materials

CMC and glycerol (analytical grade) were purchased from Nihon Shiyaku Industries Ltd. (Taipei, Taiwan). Potato peel starch (PPS) (starch content (11.52%) from dry potato peel; amylose content: 29.49% (higher than potato starch: 20.5%, corn and wheat starch: 24% [15]); and moisture content: 18.81%) was obtained by water extraction using Milli-Q water (Merck Millipore Water System, Darmstadt, Germany) in the laboratory. Seawater was obtained from Cijin Island Beach, Kaohsiung, Taiwan, and natural soil was obtained from the National Pingtung University of Science and Technology (NPUST), Pingtung, Taiwan.

### 2.2. Edible Film Production

Edible films were prepared following the method described by Oun and Rhim and Abdillah and Charles [32,33] with modifications. A response surface method (RSM) using the Central Composite Design (Design Expert ver. 13, StatEase Inc., Minneapolis, MN, USA) was employed to develop optimum PPS concentrations based on preliminary screening of the films. Resins were prepared in 100 mL beakers using RSM-optimized ratios of PPS. The ratio of 4% *w*/*v* as sample C was suggested as an optimum concentration of PPS; thus, two-fold up and down levels were used to define lower PPS (2% *w*/*v*) as sample B and higher PPS (6% (*w*/*v*)) as sample D to produce biodegradable films, and glycerol (27% *v*/*w* of PPS) was used as a plasticizer (Tables S1–S4); and CMC (2% (*w*/*v*)) (control) was mixed with glycerol (30% *v*/*v* of CMC). The resins were heated for 15 min at 100 °C on a hotplate with constant stirring, then cooled for 5 min at room temperature (25 °C). Then, the cooled resin was cast into 9 cm (diameter) Petri dishes and dried in a preheated hot air oven (DOS- 45, Deng Yng, Taipei, Taiwan) at 50 °C for 4 h. The dried samples were stored in a desiccator with silica gel, which was kept in a digital humidity controller (50% RH) for 48 h prior to analysis.

### 2.3. Visual Appearance

For the evaluation of edible film appearance, edible films were photographed using a Canon EOS 600D, Sutter speed (1/60), focus 5.6, and lenses 18.55 mm.

### 2.4. Thickness

Film thickness was measured by a digital micrometer (Syntek, Taipei, Taiwan), with a precision of ±0.001 mm, at 5 random locations. 

### 2.5. Water Solubility

Water solubility (WS) of edible films was determined according to the method described by de Faria Arquelau et al. [34], with slight modifications. Samples (3 cm × 2 cm) were oven dried at 105 °C for 24 h, cooled (30 min) in a digital humidity controller (RH = 50%), then weighed (initial weight). The samples were soaked in a 100 mL beaker with distilled water (50 mL) and continuously stirred with slow agitation (50 rpm) for 24 h at 25 °C in an isothermal reciprocal water bath shaker (SB 302, Double Eagle Enterprises Ltd., New Taipei City, Taiwan). Then, the undissolved pieces were carefully filtered out, oven dried at 105 °C for 24 h, cooled, and weighed (final weight). The experiment was conducted in triplicate, and WS was calculated according to Equation (1).

(1) $$Water\;Solubility\;\left(\%\right)=\left[\left(\frac{Final\;weight\;\left(g\right)-Initial\;weight\;\left(g\right)}{Initial\;weight\;\left(g\right)}\right)\times 100\right]$$

### 2.6. Moisture Content

The moisture content (MC) of the films was determined according to the method of Pérez-Vergara et al. [35], with slight modifications. All samples (3 cm × 2 cm) were weighed to obtain the initial weight. The samples were dried at 105 °C for 24 h, cooled in a digital humidity controller (RH = 50%) for 30 min, and then weighed (final weight). The moisture content measurements were determined in triplicate and calculated according to Equation (2).

(2) $$Moisture\;content\;\left(\%\right)=\left[\left(\frac{Final\;weight\;\left(g\right)-Initial\;weight\;\left(g\right)}{Initial\;weight\;\left(g\right)}\right)\times 100\right]\;$$

### 2.7. Swelling Power

The swelling power was determined following the procedure of Daza et al. [36]. The swelling power test was repeated in triplicate and was determined according to Equation (3).

(3) $$Swelling\;Power\;\%=\left[\left(\frac{Final\;weight\left(g\right)-Initial\;weight\left(g\right)}{Initial\;weight\;\left(g\right)}\right)\times 100\;\;\right]\;$$

### 2.8. Water Vapor Permeability 

Water vapor permeability (WVP) of edible films was evaluated following the procedure of Abdillah and Charles [32]. WVP was conducted in triplicate for all four samples and calculated according to Equation (4).

(4) $$WVP\left(g,m,\;s^{-1},\;Pa^{-1},m^{-2}\right)=\left[\left(\frac{W}{A\times t\times ∆P}\right)\times T\right]$$

where W = weight gain of the bottle (g) at time t (h); T = film thickness (m); A = exposed area of film (m^2^); ΔP = vapor pressure difference across film (Pa), calculated based on the chamber temperature and the RH inside and outside the cup.

### 2.9. Opacity

The opacity of the films was evaluated using a method proposed by da Silva Filipini et al. [6]. Edible film samples (1 cm × 4 cm) were inserted in a 1 cm quartz cuvette and analyzed in a DU^®^ 730 UV/V is spectrophotometer (Beckman- Coulter, Brea, California, United State of America) at 600 nm. The assay was repeated in triplicate, and the values were calculated using Equation (5).

(5) $$Opacity=\left[\frac{Absorbance\;at\;600\;\left(nm\right)}{Thickness\;of\;film\;\left(mm\right)}\right]\;$$

### 2.10. Mechanical Properties

The PPS films were cut into strips (25 mm × 7 mm), and then, the tensile strength (TS) and elongation at break (EAB) were measured in triplicate at a crosshead speed of 5 mm/min with 50 mm grip separation using a Tensile Machine (YD-TA Jing Koou Enterprice, Kaohsiung, Taiwan) [37].

### 2.11. Thermogravimetric Analysis 

The thermogravimetric analysis (TGA) of the films was evaluated by adapting the method used by Abdillah and Charles [32], and the thermal stabilities were characterized using a thermogravimetric analyzer (PerkinElmer, Massachusetts, Taiwan). Each film sample (~7 mg) was weighed in aluminum sample pans and heated under nitrogen gas from 25 to 550 °C, with a heating rate of 10 °C/min.

### 2.12. Differential Scanning Calorimetry 

The thermal properties (differential scanning calorimetry, DSC) of the films were determined using a DSC (PerkinElmer), as previously described by Wang et al. [38]. Each film was weighed (~7 mg), hermetically sealed in an aluminum pan using an inverted lid configuration of the DSC equipment, and heated from 25 to 210 °C at a heating rate of 10 °C/min.

### 2.13. X-ray Diffraction 

X-ray diffraction (XRD) was used following the method of Wang et al. [38], with some modifications, to determine the crystallinity of the films using an X-ray diffractometer (Bruker D8 Advance, Karlsruhe, Germany) analyzer, operating at 40 kV and 40 mA. Each sample was secured on a circular clamp of the instrument and inspected from 5 to 40 ºC. The crystallinity percentages for the edible films were calculated using Equation (6).

(6) $$Crystallinity\;\left(\%\right)=\left[\left(\frac{Area\;of\;crystalline\;peak}{Area\;of\;all\;peaks\;\left(crystaline\;and\;amorphous\right)}\right)\times 100\right]\;$$

### 2.14. Film Morphology

Surface and cross-section morphologies were determined using a scanning electron microscope (Hitachi S–300 N, Tokyo, Japan,), following the method described by da Silva Filipini et al. [6], with slight modifications. For cross-sectional topography, a small piece of the film was first attached to staples, fixed lengthwise on an aluminum stub. For surface topography, an insignificant part of the film sample was fixed onto an aluminum stub. The fixing and attachment were accomplished using a double-sided adhesive tape. Subsequently, the fixed samples were coated with a thin layer of gold at a speed of 180 s and subjected to an electronic beam, accelerating at a voltage of 10 kV. A field that represented each sample well was selected and captured at 500×.

### 2.15. Seawater and Soil Biodegradability

The biodegradability of the films was evaluated according to the method previously described by da Silva Filipini et al. [6], with modifications. Film samples (2 cm × 2 cm) were placed within a folded fabric net, which was submerged in seawater (30 mL) and kept at 26 °C. The fabric net containing the film samples was observed for degradation. In the soil tests, the film samples (2 cm × 2 cm) were buried in paper cups filled with natural soil to a depth of 4 cm. The samples were placed in a roof garden and sprayed with water (30 mL) every two days to simulate a natural soil ecosystem. The degradation progress of both experiments was evaluated at a 10-day interval; then, the film samples were photographed using a Samsung J5 camera (13 megapixels) until complete degradation.

### 2.16. Statistical Analysis

The quantitative results (thickness, water solubility, moisture content, swelling power, water vapor permeability, opacity, and mechanical properties) are expressed as mean ± standard deviation (SD). Statistical analysis was analyzed using the analysis of variance (ANOVA) and the Tukey test at *p* < 0.05 significance level using IBM^®^ SPSS^®^ Statistics version 26.0 (SPSS Inc., New York, NY, USA). 

## 3. Results and Discussion 

### 3.1. Visual Appearance and Thickness

**Figure 1:**
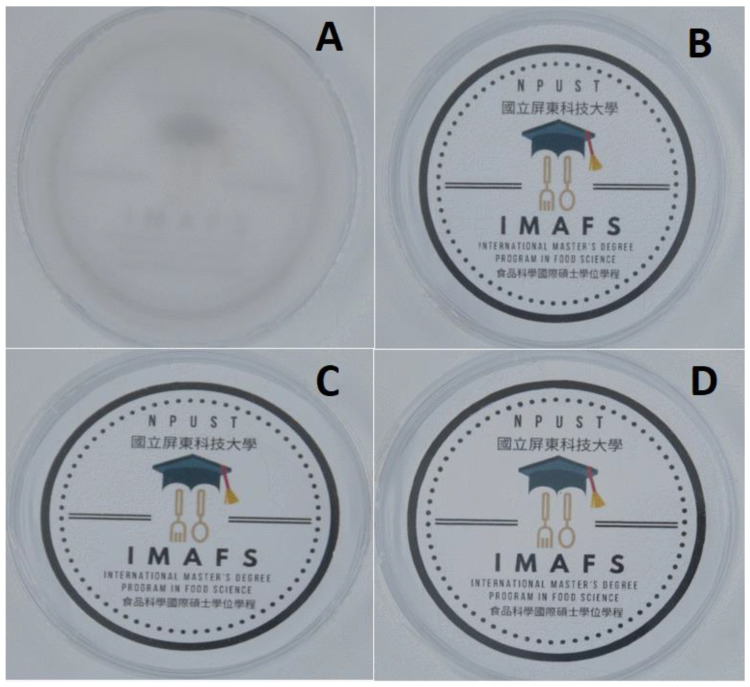
Visual appearance of the edible films: (**A**) (carboxymethyl cellulose 2% as Control); (**B**) (potato peel starch 2%); (**C**) (potato peel starch 4%); and (**D**) (potato peel starch 6%).

The visual appearances of the films are shown in Figure 1. The films appeared homogeneous, with smooth surfaces, without bubbles, and insoluble starch particles. Moreover, the films (Figure 1B–D) appeared to be more transparent compared to the CMC-based control film (Figure 1A).

All films depicted a thickness ranging between 0.040 mm and 0.119 mm, and the control had the significantly lowest thickness of 0.040 mm (Table 1). In this study, the increases in film thickness significantly corresponded with the increases in PPS concentration (*p* < 0.05). Similar trends were observed in significant (*p* < 0.05) increases in corn starch film thickness (0.059–0.085 mm) with the increments of corn starch (33.3–66.6% *w*/*w*) (de Faria Arquelau et al. [34]), and the arrowroot starch film thickness increased (0.026 to 0.082 mm) with starch concentrations (2.59–5.41%, mass/mass) (Nogueira et al. [27]).

### 3.2. Moisture Content

Low moisture content is desirable for films intended for food packaging use [39], since moisture content influences the texture and shelf life of foods. The control film (A) had a higher moisture content (22.27%) than PPSF, which ranged from 11.53% to 12.98% (Table 1). Tavares et al. [14] reported that CMC had a higher intramolecular moisture content, whereas Akhtar et al. [40] stated that CMC had several hydrophilic groups that increased its ability to interact with water molecules, thus resulting in increased attraction to water molecules. For example, CMC increased the water content of corn starch films, which confirmed the hydrophilicity of CMC [14]. Nonetheless, it is likely that CMC had a higher molecular weight than starch, which created more space between the molecules for binding water molecules and ultimately resulted in an increase in water content [41] compared to PPS. An increase in PPS concentration significantly reduced (*p* < 0.05) the moisture content of the edible films, which was attributed to the increased concentration of dissolved solids and the number of bonds between the molecules in the edible film solution [41]. A high PPS concentration might have increased the surface area for intra- and intermolecular interactions between starch macromolecules, thus increasing the inhibition of water retention by the edible films.

### 3.3. Water Solubility

Generally, for edible films to be considered suitable for packaging material, they must have low water solubility [42]. Furthermore, Nogueira et al. [27] reported that in order to enhance product durability and water resistance, an insoluble or low soluble film is required, particularly in liquid or aqueous food items. The study shows that the water solubility (WS) (Table 1) range displayed by the PPSF was significantly (*p* < 0.05) lower (18.86% to 31.06%) compared to the control group (98.90%). Furthermore, Tavares et al. [14] reported that water uptake by CMC films interacted more with water molecules than native cassava starch film, which meant that, hypothetically, PPS-based films might have fewer hydrophilic properties that limited their interaction with water molecules than the control. In addition, the films’ solubility was significantly (*p* < 0.05) reduced as the starch concentration increased and was linked to high PPS concentration, which created a highly cross-linked system that hindered water molecules from penetrating the edible film matrix system [38].

### 3.4. Swelling Power

Swelling power was significantly (*p* < 0.05) higher (521.31%) in the control film than the PPS films (Table 1) and corresponded to their higher moisture content (Table 1), which was attributed to CMC carboxyl and hydroxyl groups’ affinity for water. Similarly, in a previous study, CMC-based films exhibited higher swelling power (603%) than rice-starch-based films (85%) [43]. Edible films of high quality generally exhibit low swelling power, a phenomenon exhibited by PPSF (450.32% to 304.29%), which decreased with increments of PPS. The low swelling power of PPSF confirmed its highly cross-linked system created by the starch increments, which reduced the interaction between the film starch component and water.

### 3.5. Water Vapor Permeability

Technically, low water vapor permeability (WVP) indicates the film’s ability to limit moisture transfer between the environment and the packaged food [44,45]. The WVP of the films (Table 1) indicated that the control (A) exhibited a significantly (*p* < 0.05) higher WVP (0.061 g mm/m^2^ day KPa) (which confirmed its hydrophilicity) compared to the optimum-starch (C) and high-starch (D) concentrated PPSF (0.49 and 0.50 g mm/m^2^ day KPa, respectively) but a significantly (*p* < 0.05) lower WVP compared to low-starch (B) film (0.260 g mm/m^2^ day KPa) (Table 1). In contrast, the WVP decreased as PPS components increased, confirming the films’ increased impermeability and hydrophobicity, a trend also observed in decreased WVP (6.73 to 5.43 g mm/m^2^day KPa) as lotus starch concentrations increased from 3% to 5% [44]. Moreover, the lower WVP value indicated that the film could inhibit moisture permeating through the food product, possibly extending the shelf life of packaged foods [32].

### 3.6. Opacity

A film’s low opacity and gloss impacts the visual quality of the products, which directly influences the consumers’ purchasing preference [46], which means the higher the opacity, the lower the film’s transparency [47]. In this study, the control exhibited the highest opacity (1.50 A/mm) compared to PPSF (0.42 to 0.59 A/mm) (Table 1), which was consistent with the films’ visual appearance (Figure 1). In Domen-López et al. [15], higher transparency was exhibited by films developed from potato starch with higher lipid content compared to wheat and corn, which formed more opalescent films. Hypothetically, the lower the starch content or film thickness, the higher the amount of UV light transmitted. According to Loo and Sarbon [48], bulky films tend to scatter more light and reduce transparency. Among the PPS-based films, film B was the most opalescent, while film opacity linearly increased with PPS content.

### 3.7. Mechanical Properties

The mechanical properties of PPS films are described in Table 2. The tensile strength (TS) of films was significantly different (*p* < 0.05), with the highest TS shown by sample A (58.8 MPa), followed by sample C (13.9 MPa), sample D (10.3 MPa), and sample B (9.2 MPa). In this study, the optimum concentration of PPS (sample C) presented higher TS compared to low PPS concentration (sample A) and high PPS concentration (sample D). Comparatively, the CMC-based film (sample A) exhibited elongation at break (EAB) at 38.4%, and all PPS-based films presented lower values (15–22%). Although the TS and EAB of the PPS-based film were lower than the CMC-based films (sample A), the TS results were higher than the potato washing slurries starch film (4.28 MPa) and showed the same value with the potato starch/gelatin film (2–22 MPa); in contrast, the EAB of PPS-based films depicted lower values than the potato starch/gelatin film (60–201%) but higher than the potato washing slurries starch film (6.61%) [13,49].

### 3.8. Thermogravimetric Analysis

**Figure 2:**
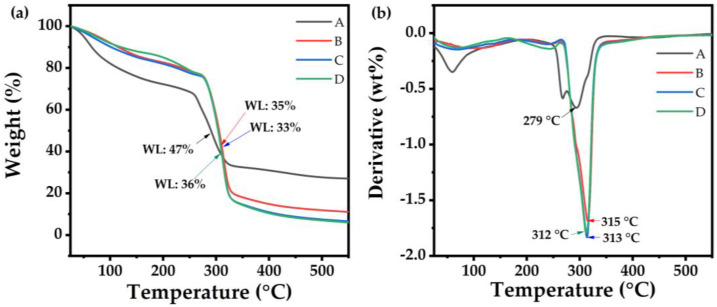
Thermogravimetric analysis (TGA) (**a**) and differential thermogravimetric analysis (DTGA) (**b**) of edible films: A (carboxymethyl cellulose 2% as Control); B (potato peel starch 2%); C (potato peel starch 4%); and D (potato peel starch 6%).

Thermal properties indicate the heating and cooling transitions of packaging materials, particularly during freezing and pasteurization. TGA (Figure 2a) and differential thermogravimetric (DTG) (Figure 2b) curves exhibited decreasing weight patterns and maximum decomposition temperatures of the PPSF. The thermal decomposition of the films occurred in three stages (Figure 2a,b), according to Suriyatem et al. [43]. The first two stages were related to the vaporization of water molecules and glycerol (35 to 150 °C). The final stage (279–315 °C) corresponded to the degradation of carbonaceous residues formed during the second stage, which occurred with the complete oxidation of these materials. The disintegration trend was similar in all the samples, although a significant shift toward lower temperatures was observed in the PPSF; hence, PPSF recorded lower weight loss than the control film. PPSF started to disintegrate between 313 and 315 °C, while the control film disintegrated at 279 °C. At about 550 °C, the final weight, which corresponded with the release process of the minerals and char residue, presented the stability of the films. As a result, low, optimum, and high concentrated PPSF displayed lower weight losses (~35.6%, 33.7%, and 36.4%, respectively) than the control (47.7%), indicating their higher thermal stabilities. Similar findings were observed when rice starch concentrations were increased in composite CMC/rice-starch-based films [43]. Nevertheless, Qin et al. [50] claim that for films to be recognized as suitable for food packaging material, they must exhibit stability when environmental temperatures are lower than 100 °C; hence, all edible films were thermally stable and could potentially be used as packaging material for perishable and/or cooled foods during storage and transportation.

### 3.9. Differential Scanning Calorimetry

**Figure 3:**
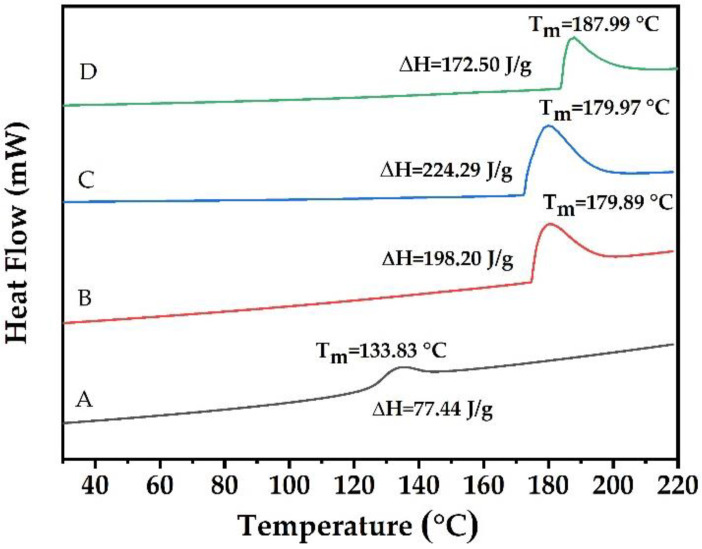
Differential scanning calorimetry (DSC) of edible films: A (carboxymethyl cellulose 2% as Control); B (potato peel starch 2%); C (potato peel starch 4%); and D (potato peel starch 6%). T_m_: melting temperature.

The thermograms (DSC) of all PPSF samples displayed an endothermic process between 20 to 210 °C, respectively (Figure 3). The thermograms of CMC films and PPSF displayed single endothermic peaks, which were attributed to the major polymers present in the films. In addition, the thermograms of CMC-based films exhibited lower glass transition, a phenomenon similarly reported for CMC films by Suriyatem et al. [43]. The control film (A) recorded the lowest T_m_, whereas PPSF (B, C, and D), demonstrated increases in T_m_ values with increments of PPS that were attributed to the hydroxyl group of PPS, which might have induced increased hydrogen bonding between the film matrix systems [48]. However, all the films produced endothermic peaks and T_m_ greater than 100 °C, which indicated that the PPSF failed to exhibit melting during thermal treatment and confirmed its potential use as packaging material in a wide range of food products. Accordingly, de Lima Barizao et al. [51] suggested that thermograms of films with a high starch concentration could result in and exhibit better thermal stable behavior.

### 3.10. Crystallinity (X-ray Diffraction)

**Figure 4:**
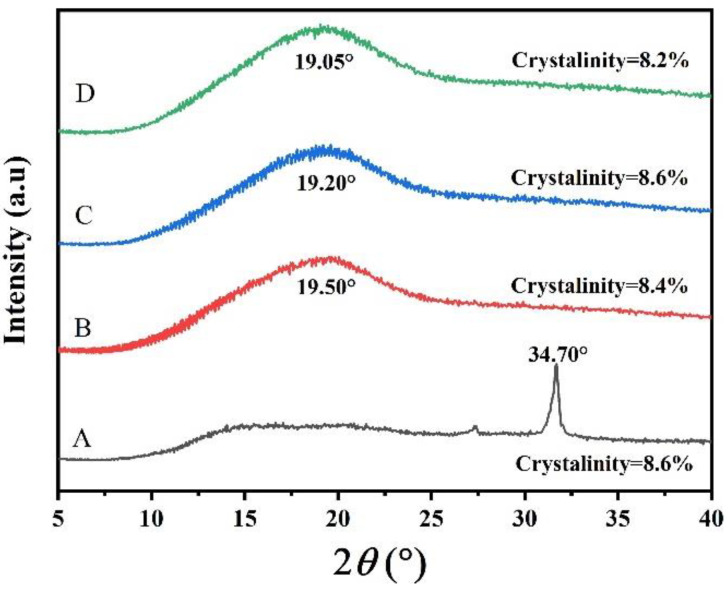
X-ray diffraction (XRD) of edible films: A (carboxymethyl cellulose 2% as Control); B (potato peel starch 2%); C (potato peel starch 4%); and D (potato peel starch 6%).

The control group depicted a narrow crystalline peak observed at 2θ = 34.7° and crystalline fractions (A, 8.6%) (Figure 4), whereas PPSF displayed broad crystalline peaks at around 2θ = 19.05° (B), 19.20° (C), and 19.50° (D) (Figure 4). The PPSF exhibited similar diffractograms and depicted amorphous characteristics with small crystalline fractions (8.4% (B), 8.6% (C), and 8.2% (D)), which were marginally different and interpreted as crystallinity being unaffected by PPS. The amorphous character of the starch films was likely induced during the casting process (thermal treatment), where intermolecular hydrogen bonding between starch molecules was disrupted (thereby inhibiting starch retrogradation) by glycerol (plasticizer), which increased the chain mobility of the starch molecules [52]. A previous study similarly reported that low crystallinity exhibited smooth surface structure, which was confirmed by SEM analysis [32]. Moreover, films depicting amorphous patterns are characteristically flexible, soft, and workable; hence, all films were considered suitable for application in food packaging.

### 3.11. Microstructure Properties

**Figure 5:**
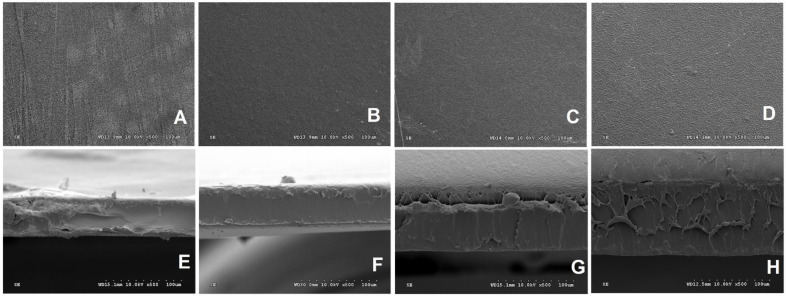
Scanning electron microscopy images of surface: (**A**) (carboxymethyl cellulose 2% as Control); (**B**) (potato peel starch 2%); (**C**) (potato peel starch 4%); and (**D**) (potato peel starch 6%). Cross-section (**E**) (carboxymethyl cellulose 2% as Control); (**F**) (potato peel starch 2%); (**G**) (potato peel starch 4%); and (**G**) (potato peel starch 6%). The magnification was 500×.

The surface section (Figure 5A–D) of all the films had no cracks, pores, and bubbles, and displayed homogenous surfaces, thus indicating the effectiveness of the casting technique. The control film (A) displayed a more opaque surface compared to PPSF, which supported the findings on opacity properties (Table 1). Moreover, the PPSF B and C appeared smooth; however, D exhibited a wrinkled surface, which indicated the higher starch concentration, which rendered the resin viscous and difficult to cast.

The cross-section micrographs (Figure 5E–G) confirmed the effect of increments of starch on film thickness demonstrated by the PPSF D sample (Table 1) and displayed heterogeneities among the films with an increase in the network of fiber-like projections as the thickness increased. Therefore, film thickness affected their cross-sectional microstructural properties, such that the thinnest among the PPSF (B) presented the least heterogeneity in the cross-sectional view, with the least network of fibers, compared to thicker edible films (Basiak et al. [53]). The microstructural properties of the edible films affected their optical properties, such that the most heterogeneous (D) film exhibited the highest opacity values, and the least heterogeneous (B) film exhibited lower opacity values. Moreover, the morphology data also supported water solubility, swelling power, and WVP reported by Santana et al. [24] (Table 1), as the structure of PPSF D appeared to be more highly complexed than other PPSF samples, which hindered water from penetrating and vapor from permeating, followed by films C and B.

### 3.12. Biodegradability

**Figure 6:**
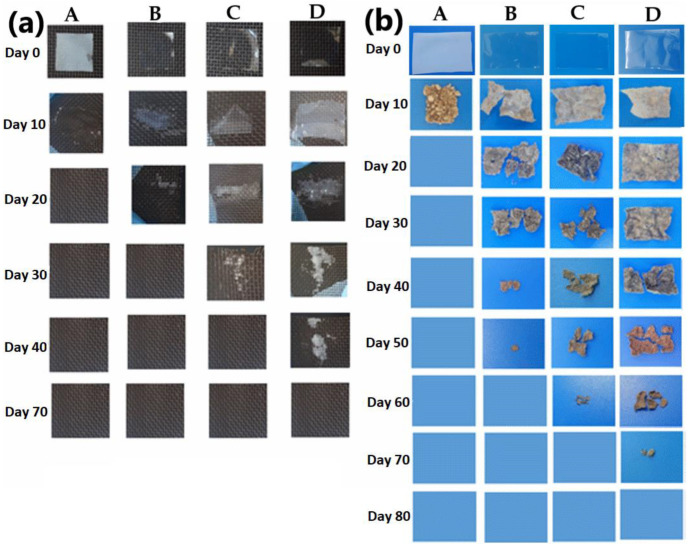
(**a**) Seawater and (**b**) soil biodegradability of edible films: A (carboxymethyl cellulose 2% as Control); B (potato peel starch 2%); C (potato peel starch 4%); and D (potato peel starch 6%).

CMC-based films and PPSF sea and soil degradation observations confirmed the biodegradation of PPSF (Figure 6a,b). The PPSF rate of biodegradation decreased with PPS increments to the films, which corresponded with their decreasing water solubility trends (Table 1). Similarly, the increments of banana starch reduced the rate of chitosan starch bioplastics, which was linked to low water-soluble films [54]. In contrast, the higher water-soluble films exhibited accelerated biodegradation from 10 to 40 days (Figure 6a) and 20 to 60 days (Figure 6b). Carissimi et al. [55] stated that high water-soluble films, such as the control film A (Table 1), tend to biodegrade faster because of their hydrophilic properties, which accelerated the degradation of the films. Fungi are reportedly responsible for soil biodegradation, whereas bacteria are dominant in the aquatic environment [56]. Based on Figure 6, the films likely degraded following the hydro-biodegradation mechanism [56], which was apparent in the more hydrophilic films A, B, and C. However, all edible films degraded within the suggested time frames, which confirmed their potential application in the food packaging industry.

## 4. Conclusions

Potato-peel-starch-based films (PPSF) depicted a highly cross-linked film matrix that exhibited improved thickness, opacity, heterogeneity of surface, and cross-sectional areas compared with CMC films. Moreover, PPS increments decreased water solubility, swelling power, water permeability and displayed good mechanical properties; however, these increments failed to affect the visual appearance and crystallinity properties of the films. PPSF demonstrated accelerated soil and seawater biodegradability and thickness within the recommended standards of ~90%: 2 years, 6 months, and ≤0.25 mm, respectively, which proved potato peel as a low-cost starch source for the production of biodegradable edible films. The bioconversion of potato peel starch to edible films could contribute toward recycling food wastes and losses by contributing to the shelf life and safety of food products, as well as decreasing the volume of petroleum-based plastics entering the environment.

## Figures and Tables

**Table 1:** Physicochemical, water barrier, and opacity properties of potato-peel-starch-based edible film ^1^.

Sample Code	T (mm)	MC (%)	WS (%)	SP (%)	WVP (g mm/m^2^ Day KPa)	O (A/mm)
A	0.040 ± 0.0015 ^d^	22.27 ± 0.44 ^a^	98.90 ± 0.17 ^a^	521.31 ± 3.49 ^a^	0.061 ± 0.001 ^b^	1.50 ± 0.062 ^a^
B	0.059 ± 0.0016 ^c^	12.92 ± 0.39 ^b^	31.06 ± 0.24 ^b^	450.32 ± 3.44 ^b^	0.260 ± 0.004 ^a^	0.42 ± 0.020 ^d^
C	0.092 ± 0.0014 ^b^	12.98 ± 0.27 ^b^	24.93 ± 0.14 ^c^	365.26 ± 3.09 ^c^	0.049 ± 0.002 ^c^	0.50 ± 0.036 ^c^
D	0.119 ± 0.0020 ^a^	11.53 ± 0.75 ^c^	18.86 ± 0.22 ^d^	304.29 ± 3.62 ^d^	0.050 ± 0.001 ^c^	0.59 ± 0.019 ^b^

^1^ Mean values in the same column with different superscript letters (a–d) are significantly different, using Tukey test (*p* < 0.05). A (carboxymethyl cellulose 2% as Control); B (potato peel starch 2%); C (potato peel starch 4%); and D (potato peel starch 6%). T: Thickness; MC: Moisture Content; WS: Water Solubility; SP: Swelling Power; WVP: Water Vapor Permeability; and O: Opacity.

**Table 2:** Mechanical properties ^1^.

Sample Code (*w*/*v*)	Tensile Strength (MPa)	Elongation at Break (%)
A	58.830 ± 8.27 ^a^	38.43 ± 6.06 ^a^
B	9.23 ± 1.13 ^b^	15.04 ± 4.34 ^b^
C	13.95 ± 4.14 ^b^	22.65 ± 8.72 ^b^
D	10.39 ± 1.05 ^b^	22.25 ± 3.97 ^b^

^1^ Mean values in the same column with different superscript letters (a–b) are significantly different using the Tukey test (*p* < 0.05). A (carboxymethyl cellulose 2% as Control); B (potato peel starch 2%); C (potato peel starch 4%); and D (potato peel starch 6%).

## Data Availability

Not applicable.

## Supplementary Materials

The following supporting information can be downloaded at: https://www.mdpi.com/article/10.3390/polym14173462/s1, Table S1: Optimization design of potato peel starch with glycerol; Table S2: Response of optimization Design of potato peel starch with glycerol; Table S3: Equation model suggestion for optimization design of potato peel starch with glycerol; Table S4: Point prediction from optimization design of potato peel starch with glycerol.

## References

[1] FAO, IFAD, WFP (2013). The State of Food Insecurity in the World, 2013: The Multiple Dimensions of Food Security.

[2] FAO (2011). Global Food Losses and Food Waste: Extent, Causes and Prevention.

[3] FAO (2019). The State of Food and Agriculture 2019. Moving Forward on Food Loss and Waste Reduction.

[4] Panda P.K., Dash P., Yang J.M., Chang Y.H. (2022). Development of Chitosan, Graphene Oxide, and Cerium Oxide Composite Blended Films: Structural, Physical, and Functional Properties. Cellulose.

[5] Ahmed S. (2018). Bio-Based Materials for Food Packaging: Green and Sustainable Advanced Packaging Materials.

[6] da Silva Filipini G., Romani V.P., Martins V.G. (2020). Biodegradable and Active-Intelligent Films Based on Methylcellulose and Jambolão (*Syzygium Cumini*) Skins Extract for Food Packaging. Food Hydrocoll..

[7] Aguirre-Joya J.A., De Leon-Zapata M.A., Alvarez-Perez O.B., Torres-León C., Nieto-Oropeza D.E., Ventura-Sobrevilla J.M., Aguilar M.A., Ruelas-Chacón X., Rojas R., Ramos-Aguiñaga M.E. (2018). Basic and Applied Concepts of Edible Packaging for Foods. Food packaging and preservation.

[8] Jiang T., Duan Q., Zhu J., Liu H., Yu L. (2020). Starch-Based Biodegradable Materials: Challenges and Opportunities. Adv. Ind. Eng. Polym. Res..

[9] Panda P.K., Sadeghi K., Seo J. (2022). Recent Advances in Poly ( Vinyl Alcohol )/ Natural Polymer Based Films for Food Packaging Applications: A Review. Food Packag. Shelf Life.

[10] Niu X., Wang W., Kitamura Y., Wang J., Sun J., Ma Q. (2021). Design and Characterization of Bio-Amine Responsive Films Enriched with Colored Potato (Black King Kong) Anthocyanin for Visual Detecting Pork Freshness in Cold Storage. J. Food Meas. Charact..

[11] Azmin S.N.H.M., Hayat N.A.B.M., Nor M.S.M. (2020). Development and Characterization of Food Packaging Bioplastic Film from Cocoa Pod Husk Cellulose Incorporated with Sugarcane Bagasse Fibre. J. Bioresour. Bioprod..

[12] Thakur R., Saberi B., Pristijono P., Golding J., Stathopoulos C., Scarlett C., Bowyer M., Vuong Q. (2016). Characterization of Rice Starch-ι-Carrageenan Biodegradable Edible Film. Effect of Stearic Acid on the Film Properties. Int. J. Biol. Macromol..

[13] Niu X., Ma Q., Li S., Wang W., Ma Y., Zhao H., Sun J., Wang J. (2021). Preparation and Characterization of Biodegradable Composited Films Based on Potato Starch/Glycerol/Gelatin. J. Food Qual..

[14] Tavares K.M., de Campos A., Mitsuyuki M.C., Luchesi B.R., Marconcini J.M. (2019). Corn and Cassava Starch with Carboxymethyl Cellulose Films and Its Mechanical and Hydrophobic Properties. Carbohydr. Polym..

[15] Domene-López D., Delgado-Marín J.J., Martin-Gullon I., García-Quesada J.C., Montalbán M.G. (2019). Comparative Study on Properties of Starch Films Obtained from Potato, Corn and Wheat Using 1-Ethyl-3-Methylimidazolium Acetate as Plasticizer. Int. J. Biol. Macromol..

[16] Philp J. (2014). OECD Policies for Bioplastics in the Context of a Bioeconomy, 2013. Ind. Biotechnol..

[17] Purwanti T.S., Syafrial S., Huang W.C., Saeri M. (2022). What Drives Climate Change Adaptation Practices in Smallholder Farmers? Evidence from Potato Farmers in Indonesia. Atmosphere.

[18] Biswal A.K., Panda P.K., Yang J.M., Misra P.K. (2020). Isolation, Process Optimisation and Characterisation of the Protein from the de-Oiled Cake Flour of Madhuca Latifolia. IET Nanobiotechnol..

[19] Javed A., Ahmad A., Tahir A., Shabbir U., Nouman M., Hameed A. (2019). Potato Peel Waste-Its Nutraceutical, Industrial and Biotechnological Applacations. AIMS Agric. Food.

[20] Matheson M.T. (2019). Disposal Is Not Free: Fiscal Instruments to Internalize the Environmental Costs of Solid Waste.

[21] Ma Y., Zhao H., Ma Q., Cheng D., Zhang Y., Wang W., Wang J., Sun J. (2022). Development of Chitosan/Potato Peel Polyphenols Nanoparticles Driven Extended-Release Antioxidant Films Based on Potato Starch. Food Packag. Shelf Life.

[22] Ferreira M.S.L., Fai A.E.C., Andrade C.T., Picciani P.H., Azero E.G., Gonçalves É.C.B.A. (2016). Edible Films and Coatings Based on Biodegradable Residues Applied to Acerolas (*Malpighia Punicifolia* L.). J. Sci. Food Agric..

[23] Gebrechristos H.Y., Ma X., Xiao F., He Y., Zheng S., Oyungerel G., Chen W. (2020). Potato Peel Extracts as an Antimicrobial and Potential Antioxidant in Active Edible Film. Food Sci. Nutr..

[24] Santana R.F., Bonomo R.C.F., Gandolfi O.R.R., Rodrigues L.B., Santos L.S., dos Santos Pires A.C., de Oliveira C.P., da Costa Ilheu Fontan R., Veloso C.M. (2018). Characterization of Starch-Based Bioplastics from Jackfruit Seed Plasticized with Glycerol. J. Food Sci. Technol..

[25] Khan B., Niazi M.B.K., Jahan Z., Farooq W., Naqvi S.R., Ali M., Ahmed I., Hussain A. (2019). Effect of Ultra-Violet Cross-Linking on the Properties of Boric Acid and Glycerol Co-Plasticized Thermoplastic Starch Films. Food Packag. Shelf Life.

[26] Gordillo C.A.S., Valencia G.A., Zapata R.A.V., Henao A.C.A. (2014). Physicochemical Characterization of Arrowroot Starch (Maranta Arundinacea Linn) and Glycerol/Arrowroot Starch Membranes. Int. J. Food Eng..

[27] Nogueira G.F., Fakhouri F.M., de Oliveira R.A. (2018). Extraction and Characterization of Arrowroot (Maranta Arundinaceae L.) Starch and Its Application in Edible Films. Carbohydr. Polym..

[28] Kaur G., Sharma S., Mir S.A., Dar B.N. (2021). Nanobiocomposite Films: A “Greener Alternate” for Food Packaging. Food Bioprocess Technol..

[29] Zhao L., Duan G., Zhang G., Yang H., Jiang S., He S. (2020). Electrospun Functional Materials toward Food Packaging Applications: A Review. Nanomaterials.

[30] Mohamed S.A.A., El-Sakhawy M., El-Sakhawy M.A.-M. (2020). Polysaccharides, Protein and Lipid —Based Natural Edible Films in Food Packaging: A Review. Carbohydr. Polym..

[31] Ezati P., Riahi Z., Rhim J.W. (2022). CMC-Based Functional Film Incorporated with Copper-Doped TiO2 to Prevent Banana Browning. Food Hydrocoll..

[32] Abdillah A.A., Charles A.L. (2021). Characterization of a Natural Biodegradable Edible Film Obtained from Arrowroot Starch and Iota-Carrageenan and Application in Food Packaging. Int. J. Biol. Macromol..

[33] Oun A.A., Rhim J.-W. (2015). Preparation and Characterization of Sodium Carboxymethyl Cellulose/Cotton Linter Cellulose Nanofibril Composite Films. Carbohydr. Polym..

[34] de Faria Arquelau P.B., Silva V.D.M., Garcia M.A.V.T., de Araújo R.L.B., Fante C.A. (2019). Characterization of Edible Coatings Based on Ripe “Prata” Banana Peel Flour. Food Hydrocoll..

[35] Pérez-Vergara L.D., Cifuentes M.T., Franco A.P., Pérez-Cervera C.E., Andrade-Pizarro R.D. (2020). Development and Characterization of Edible Films Based on Native Cassava Starch, Beeswax, and Propolis. NFS J..

[36] Daza L.D., Homez-Jara A., Solanilla J.F., Váquiro H.A. (2018). Effects of Temperature, Starch Concentration, and Plasticizer Concentration on the Physical Properties of Ulluco (Ullucus Tuberosus Caldas)-Based Edible Films. Int. J. Biol. Macromol..

[37] Abdillah A.A., Lin H., Charles A.L. (2022). International Journal of Biological Macromolecules Development of Halochromic Indicator Film Based on Arrowroot Starch / Iota-Carrageenan Using Kyoho Skin Extract to Monitor Shrimp Freshness. Int. J. Biol. Macromol..

[38] Wang K., Wang W., Ye R., Liu A., Xiao J., Liu Y., Zhao Y. (2017). Mechanical Properties and Solubility in Water of Corn Starch-Collagen Composite Films: Effect of Starch Type and Concentrations. Food Chem..

[39] Ahmad M., Benjakul S., Prodpran T., Agustini T.W. (2012). Physico-Mechanical and Antimicrobial Properties of Gelatin Film from the Skin of Unicorn Leatherjacket Incorporated with Essential Oils. Food Hydrocoll..

[40] Akhtar H.M.S., Riaz A., Hamed Y.S., Abdin M., Chen G., Wan P., Zeng X. (2018). Production and Characterization of CMC-Based Antioxidant and Antimicrobial Films Enriched with Chickpea Hull Polysaccharides. Int. J. Biol. Macromol..

[41] Hatmi R.U., Apriyati E., Cahyaningrum N. (2020). Edible Coating Quality with Three Types of Starch and Sorbitol Plasticizer. Proceedings of the E3S Web of Conferences.

[42] Ulfah M., Salsabila A., Rohmawati I. (2018). Characteristics of Water Solubility and Color on Edible Film from Bioselulosa Nata Nira Siwalan with the Additional of Glycerol. Proceedings of the International Conference on Mathematics, Science and Education 2017 (ICMSE2017).

[43] Suriyatem R., Auras R.A., Rachtanapun P. (2019). Utilization of Carboxymethyl Cellulose from Durian Rind Agricultural Waste to Improve Physical Properties and Stability of Rice Starch-Based Film. J. Polym. Environ..

[44] Sukhija S., Singh S., Riar C.S. (2016). Analyzing the Effect of Whey Protein Concentrate and Psyllium Husk on Various Characteristics of Biodegradable Film from Lotus (*Nelumbo Nucifera*) Rhizome Starch. Food Hydrocoll..

[45] Oyeoka H.C., Ewulonu C.M., Nwuzor I.C., Obele C.M., Nwabanne J.T. (2021). Packaging and Degradability Properties of Polyvinyl Alcohol/Gelatin Nanocomposite Films Filled Water Hyacinth Cellulose Nanocrystals. J. Bioresour. Bioprod..

[46] Nawab A., Alam F., Haq M.A., Lutfi Z., Hasnain A. (2017). Mango Kernel Starch-Gum Composite Films: Physical, Mechanical and Barrier Properties. Int. J. Biol. Macromol..

[47] Lozano-Navarro J.I., Díaz-Zavala N.P., Velasco-Santos C., Martínez-Hernández A.L., Tijerina-Ramos B.I., García-Hernández M., Rivera-Armenta J.L., Páramo-García U., Reyes-de la Torre A.I. (2017). Antimicrobial, Optical and Mechanical Properties of Chitosan–Starch Films with Natural Extracts. Int. J. Mol. Sci..

[48] Loo C.P.Y., Sarbon N.M. (2020). Chicken Skin Gelatin Films with Tapioca Starch. Food Biosci..

[49] Romeira K.M., Abdalla G., Gonçalves R.P., Pegorin G.S., de Azeredo H.M.C., Mussagy C.U., Herculano R.D. (2021). Residual Starch Packaging Derived from Potato Washing Slurries to Preserve Fruits. Food Bioprocess Technol..

[50] Qin Y., Liu Y., Yuan L., Yong H., Liu J. (2019). Preparation and Characterization of Antioxidant, Antimicrobial and PH-Sensitive Films Based on Chitosan, Silver Nanoparticles and Purple Corn Extract. Food Hydrocoll..

[51] de Lima Barizão C., Crepaldi M.I., Oscar de Oliveira S., de Oliveira A.C., Martins A.F., Garcia P.S., Bonafé E.G. (2020). Biodegradable Films Based on Commercial κ-Carrageenan and Cassava Starch to Achieve Low Production Costs. Int. J. Biol. Macromol..

[52] Hornung P.S., Avila S., Masisi K., Malunga L.N., Lazzarotto M., Schnitzler E., Ribani R.H., Beta T. (2018). Green Development of Biodegradable Films Based on Native Yam (Dioscoreaceae) Starch Mixtures. Starch-Stärke.

[53] Basiak E., Lenart A., Debeaufort F. (2017). Effect of Starch Type on the Physico-Chemical Properties of Edible Films. Int. J. Biol. Macromol..

[54] Sapei L., Padmawijaya K.S., Sijayanti O., Wardhana P.J. (2015). The Effect of Banana Starch Concentration on the Properties of Chitosan-Starch Bioplastics. J. Chem. Pharm. Res..

[55] Carissimi M., Flôres S.H., Rech R. (2018). Effect of Microalgae Addition on Active Biodegradable Starch Film. Algal Res..

[56] Hodzic A., Baillie C. (2004). Re-Use, Recycling and Degradation of Composites. Green Composites.

