# Supplementary material for: A Comprehensive Characterization of Biodegradable Edible Films Based on Potato Peel Starch Plasticized with Glycerol
Source: Polymers (Basel). 2022 Aug 25;14(17):3462. doi: 10.3390/polym14173462 (PMC9460672; doi:10.3390/polym14173462)
Supplement: Supplementary file 1 [file polymers-14-03462-s001.zip › polymers-1877436-supplementary.pdf]

Supplementary data

A Comprehensive Characterization of Biodegradable Edible Films Based on Potato Peel Starch Plasticized with Glycerol

[1] Department of Tropical Agriculture and International Cooperation, National Pingtung University of Science and Technology,1 Shuefu Road, Neipu, Pingtung 91201, Taiwan

[2] International Master Degree Program in Food Science, National Pingtung University of Science and Technology,1 Shuefu Road, Neipu, Pingtung 91201, Taiwan

[3] Department of Marine, Faculty of Fisheries and Marine, Universitas Airlangga, Campus C UNAIR, Mulyorejo, Surabaya 60115 , Indonesia

*Correspondence: alcharles@mail.npust.edu.tw

Table S1. Optimization design of potato peel starch with glycerol

	Variable			Response
Run	Temperature (°C)	Starch (%)	Plasticizer 9%)	Thickness (mm)
1	60	4	30	0.08
2	70	5	35	0.1
3	50	5	35	0.1
4	60	4	30	0.09
5	60	4	30	0.09
6	60	4	21.591	0.08
7	70	5	25	0.1
8	60	4	30	0.09
9	60	4	30	0.09
10	70	3	25	0.05
11	70	3	35	0.06
12	50	5	25	0.1
13	60	5.68	30	0.11
14	76.82	4	30	0.09
15	60	2.3821	30	0.05
16	50	3	25	0.05
17	60	4	38.409	0.09
18	43.1821	4	30	0.09
19	50	3	35	0.06
20	60	4	30	0.09

Table S2. Response of optimization Design of potato peel starch with glycerol

Response	Model	Regression	F-Model/Lack of fit	p Value model/lack of fit	Model/Lack of fit
Thickness(mm)	Linear	0.9364	78.48/1.38	<0.0001/0.3808	Significant/ not significant

Table S3. Equation model suggestion for optimization design of potato peel starch with glycerol

Response	Equation[1]
Thickness (mm)	0.0813−0.000009.985A+0.0193 B+0.0012 C

[1]A; Temperature; B: Starch; C: Plasticizer

Table S4. Point prediction from optimization design of potato peel starch with glycerol

Temperature (°C)	Starch (%)	Plasticizer (%)	Thickness (mm)
51.986	4.48	26.52	0.096
